# Supplementary material for: Challenges Addressing Inequalities in Measles Vaccine Coverage in Zambia through a Measles–Rubella Supplementary Immunization Activity during the COVID-19 Pandemic
Source: Vaccines (Basel). 2023 Mar 7;11(3):608. doi: 10.3390/vaccines11030608 (PMC10059977; doi:10.3390/vaccines11030608)
Supplement: Supplementary file 1 [file vaccines-11-00608-s001.zip › vaccines-2252053-supplementary.pdf]

Supplementary Material

Title: Challenges Addressing Inequalities in Measles Vaccine Coverage in Zambia through a Measles–Rubella Supplementary Immunization Activity during the COVID-19 Pandemic

Table S1: Measles and rubella vaccination coverage (at least one dose) among children between 9 months - 59 months.

Ever vaccinated against measles	Unweighted N (%)	Weighted % (95% CI)
**Estimated routine measles and rubella immunization coverage before the campaign**		
Documented/card verified	2169 (46.7)	47.1 (45.5, 48.6)
Recall (positive) only	1762 (38.0)	37.8 (36.3, 39.4)
Card+positive recall	3931 (84.7)	84.9 (81.8, 88.0)
Unvaccinated	709 (15.3)	15.1 (13.6, 16.8)
**SIA**		
Documented/card verified	113 (2.4)	2.7 (2.2, 3.2)
Recall (positive) only	3073 (66.2)	65.9 (64.5, 67.4)
Card+positive recall	3186 (68.6)	68.6 (66.7, 70.6)
Unvaccinated during the campaign	1454 (31.4)	31.4 (29.8, 33.2)

**Either Routine or SIA**		
Documented/card verified	2187 (47.1)	47.5 (45.9, 49.0)
Recall (positive) only	1941 (41.8)	41.6 (40.1, 43.2)
Card+positive recall	4128 (88.9)	89.1 (86.0, 92.2)
Unvaccinated	512 (11.1)	10.9 (9.7, 12.3)

Table S2: Measles and rubella vaccination coverage (at least one dose) among children between 9 months to 59 months, stratified by 9–17 months and 18–59 months

	9-17 Months		18-59 Months	
**Ever vaccinated against measles**	**Unweighted N (%)**	**Weighted % (95% CI)**	**Unweighted N (%)**	**Weighted % (95% CI)**
**Estimated routine measles and rubella immunization coverage before the campaign**				
Documented/card verified	384 (54.0)	53.9 (49.9, 57.8)	1785 (45.4)	45.8 (44.1, 47.5)
Recall (positive) only	217 (30.6)	30.0 (26.4, 33.8)	1545 (39.3)	39.3 (37.6, 40.9)
Card+positive recall	601 (84.6)	83.9 (76.3, 91.6)	3330 (84.7)	85.7 (81.7, 86.6)
Unvaccinated	109 (15.4)	16.1 (12.3, 21.1)	600 (15.3)	14.3 (13.2, 16.8)
**SIA**				
Documented/card verified	37 (5.2)	5.6 (4.0, 7.8)	76 (1.9)	2.1 (1.7, 2.7)
Recall (positive) only	455 (64.1)	64.0 (60.1, 67.7)	2618 (66.6)	66.3 (64.7, 67.9)
Card+positive recall	492 (69.3)	69.6 (64.1, 75.5)	2694 (68.5)	68.5 (66.4, 70.6)
Unvaccinated during the campaign	218 (30.7)	30.4 (26.7, 36.1)	1236 (31.5)	31.5 (29.7, 33.5)
**Either Routine or SIA**				
Documented/card verified	394 (55.5)	55.5 (51.5, 59.4)	1793 (45.6)	46.0 (44.3, 47.7)
Recall (positive) only	240 (33.8)	33.5 (29.8, 37.4)	1701 (43.4)	43.1 (41.4, 44.8)

Card+positive recall	634 (89.3)	89.0 (81.3, 96.8)	3494 (88.9)	89.1 (85,7, 89.5)
Unvaccinated	76 (10.7)	11.0 (8.5, 15.5)	436 (11.1)	10.9 (9.6, 12.3)

Table S3: Measles and rubella vaccination coverage (at least one dose) among children between 9 months to 59 months, treating unknown as vaccinated.

Ever vaccinated against measles	Unweighted N (%)	Weighted % (95% CI)
**Estimated routine measles and rubella immunization coverage before the campaign**		
Vaccinated	4201 (90.5)	90.6 (86.8, 94.4)
Unvaccinated	439 (9.5)	9.4 (8.6, 10.4)
**SIA**		
Vaccinated	3236 (69.7)	69.7 (67.5, 72.1)
Unvaccinated during the campaign	1404 (30.3)	30.3 (29.0, 31.7)
**Either Routine or SIA**		
Vaccinated	4178 (90.0)	90.2 (86.8, 93.7)
Unvaccinated	462 (10.0)	9.8 (8.9, 10.8)

Table S4: Unweighted and weighted estimates of the number of routine and SIA MR doses that each child 9–59 months had received

	Unweighted N (%)	Weighted % (95% CI)
0 dose	534 (11.5)	10.9 (9.7, 12.3)
1 routine dose	172 (3.7)	3.9 (3.0, 4.1)
1 SIA dose	211 (4.5)	4.2 (0.9, 4.6)
2 routine doses	757 (16.3)	16.6 (15.5, 17.8)
1 routine dose + 1 SIA dose	291 (6.3)	6.3 (5.6, 7.1)
2 or more routine dose + 1 SIA dose	2675 (57.7)	58.1 (59.8, 62.8)

0 dose: children who had a documented record or recalled not having received any MCV doses though routine immunization or SIA; 1 routine dose: children who had a documented record or recalled having received MCV1 through routine immunization; 2 routine doses: children who had a documented record or recalled having received MCV1 and MCV2 through routine immunization; 1 SIA dose: children who had a documented record or recalled not having received any MCV through routine immunization and a had documented record or recalled having received MCV1 through SIA; 1 routine dose + 1 SIA dose: children who had a documented record or recalled having received MCV1 through routine immunization and had a documented record or recalled having received MCV2 through SIA; 2 or more routine doses + 1 SIA dose: children who had a documented record or recalled having received MCV1 and MCV2 and/or additional MCVs through routine immunization and recalled having received an additional MCV through SIA.

Table S5: Receipt of MR vaccine among children 9–59 months post-SIA by strategy, including only documented vaccination

	Unweighted N (%)	Weighted % (95% CI)
0 dose	95 (3.4)	3.6 (2.9, 4.4)
1 routine dose	745 (27.0)	26.5 (24.8, 28.3)
1 SIA dose	39 (1.4)	1.5 (1.0, 2.0)
2 routine doses	1234 (44.7)	45.4 (43.4, 47.5)
1 routine dose + 1 SIA dose	66 (2.4)	2.7 (2.1, 3.4)
≥1 dose with unknown source[1]	585 (21.2)	20.3 (18.7, 22.0)
Total	2672	

1. ">=1 dose with unknown source" were children had vaccination documents other than vaccination card verified, which can indicate children were vaccinated for at least one dose of MCV

Supplementary Methods

Survey Sampling Methods

1. Target population

The survey will enroll children who were eligible (9 – 59 months) for the November 2020 MR SIA. The birth date range for these children was established by subtracting 59 months for the oldest birth date and 9 months for the youngest birth date. The earliest acceptable date of birth is 23rd December 2015 and the latest is 28th February 2020.

(EDATE (date of first day of SIAs, negative number of the lowest age number = - 9))
(EDATE (date of first day of SIAs, negative number of the highest age number = - 59))

2. Sampling frames

Frames provide list from which samples are drawn. The 2010 census frame of housing will be used to draw a sample of primary sampling units (PSUs) in this survey

3. Sample size calculations

To determine the number of children to be surveyed, the following parameters will be considered:
1. The number of strata where the survey will be conducted.
2. The effective sample size (ESS).
3. The design effect (DEFF).
4. The average number of households to visit to find an eligible child.
5. An inflation factor to account for nonresponse.

The survey will have representation at regional level (rural / urban), therefore the number of strata is two (2). The sample size for rural and urban will be combined to come up with the national sample size.

To determine the effective sample size (ESS), the following parameters were considered:
Desired level of precision of coverage data
Estimate of a key indicator to be measured by the survey, in this case is is the expected immunization coverage in the survey area.
The 95% confidence interval limit

The following formula is used in calculating the effective sample size (SRS) is

$$n = \frac{\left(z^2 p(1-p)\right)}{e^2}$$

n is the required sample size
z defines the level of confidence desired. z =1.96 for a 95% CI;
p is an estimate of a key indicator (percentage) to be measured by the survey;
e is the margin of error to be attained (0.05).

To obtain survey estimates for each stratum, the following parameters were used in the design:
The number of strata within the design in which case it is 1 for every region (rural/urban)
The Effective Sample Size or simple random sample at a desired precision of $\pm$5% and expected immunization coverage.)

The design effect DEFF which was calculated by squaring the DEFT for measles vaccination per province in the Zambia Demographic Health Survey 2018

The average number of households to visit to find an eligible child. Calculate an inflation factor to account for nonresponse assumption of a 10 percent non response rate is used
Table S6 shows the required sample size of 1,463 children in 272 clusters.

Table S6: Details of survey design

Design#	Strata	#sub strata	ESS (12 - 23)	ESS (24 - 59)	ESS	Interviews per SEA	DEFT (ZHDS 2018)	DEFF	interval of hh with eligibles	Non response factor	Estimated sample size Ncs	Nhh to visit per stratum	No of clusters	N per cluster
		A			*B*	*m*		*C*	*D*	*E*	*A x B x C*	*A x B x C x D x E*	*(B x C) /m*	*D x E x m*
**1**	**Urban**	1	116	354	469	5.5	1.121	1.26	6.868	1.11	590	4,500	**110**	42
**2**	**Rural**	1	132	356	488	5.5	1.338	1.79	6.868	1.11	873	6,665	**162**	42
	**Zambia**		**248**	**709**	**957**						**1,463**	**11,165**	**272**	

4. Survey design and representation

The survey design has two explicit strata over which survey results will have representation at 95% CI. The two strata are urban Zambia (110 EAs) and rural Zambia (162 EAs). Additionally survey results will be representative at national level which is a sum of the two strata (272 clusters).

5. Sample allocation

Zambia is divided into 10 provinces, within each province, there are districts, and constituencies are found within each district. Within each constituencies, there are wards and Census Supervisory areas within each ward. Standard enumeration areas are in turn found within each ward. Because representation in this survey is required at rural urban level as well as at national level, it is imperative that the sample has a national widespread with each strata for good representation. Therefore, the sample allocation within each explicit strata will be done such that provinces within each strata will be treated as separate strata. The allocation will be done proportional to the size of the province within the respective strata. The measure of size used will be the number of households as reported in the 2010 census of housing and population.

Table S7 shows the sample allocation within provinces

Table S7: Sample allocation within provinces

Sn	Province	Urban	Rural	Total
1	Central	6	19	25
2	Copperbelt	32	7	39
3	Eastern	4	29	33
4	Luapula	4	16	20
5	Lusaka	43	8	51
6	Muchinga	3	15	18
7	Northern	5	18	23
8	North Western	3	12	15
9	Southern	8	23	31
10	Western	2	15	17
	**Total**	**110**	**162**	**272**

6. Sampling Design

The sample selection will be a two-stage sample design. In the first stage, 110 and 162 Primary Sampling Units (PSUs) will be selected from the urban and rural regions respectively. Within each strata the selection of PSU will be as detailed in table 2. Thereafter, in the second stage 42 households in each selected cluster are then going to be randomly sampled for enrolment in the survey. It is estimated that about 14.6 percent of households will have eligible survey participates aged 9 to 60 months based on the proportion of the eligible population to the

projected population. Based on this proportion, 42 households will have to be enrolled in the survey, in order to find at least 5 children per cluster. A complete listing of households will be done within the selected Enumeration Areas (EAs), to have an updated household sampling frame which will be vital to calculate accurate probabilities of selection which are essential for good survey estimates.

For each enumeration area (EA), a listing of households will be done. The listing will be done in a systematic manner and all households will be numbered serially from 1 to N. The total number of households listed will be designated as N. From each EA 20 households (where n=20) will be selected using systematic random sampling. A sampling interval will be calculated as N/n. A random number between 1 and the sampling interview will be selected using an excel spreadsheet and the RANDBETWEEN function. This function selects a rand number between one and the interval specified. The number selected will be the random start or the first household to be listed. Once first household is done, the next household will be obtained by adding an interval.

First Stage Selection

At the first sampling stage, the sampled EAs will be selected within the provinces systematically with probability proportional to size (PPS) from the ordered list of EAs on the census 2010 sampling frame. The measure of size for each EA is based on the number of households in each PSU on the 2010 Census frame. To ensure representation from the whole target area, the frame will be sorted by province, district, constituency, ward, rural/urban, CSA and SEA. This is some form of implicit stratification since these variables in the frame follow geographical ordering. The sampling procedure is given below:

(1) Sort the SEAs in the target area by province district, constituency, ward, rural/urban, CSA and SEA

(2) Cumulate the measures of size (number of households) down the ordered list of SEAs within strata. The final cumulated measure of size will be the total number of households in the frame for the target area 'h' (M_h).

(3) To obtain the sampling interval for target area 'h' (I_h), divide M_h by the total number of SEAs to be selected in stratum 'h' (n_h):
$I_h = M_h / I_h$

(4) Select a random number (R_h) between 0 and I_h . The sample EAs will be identified by the following selection numbers
$S_{hi} = R_h + [I_h x(i - 1)]$, rounded up,
Were $i = 1, 2, 3 \ldots n_h$

The Microsoft Excel software will be used for selecting the sample SEAs from the sampling frame using the steps described above.

Second Stage Selection (HH selection)

The following systematic random sampling method will be used to select households:
Serially number all responding households from *1 to N* (the total number of responding households listed in the cluster)

Calculate the sampling interval ***K =N/n [Rounded up]***, where ***n*** = sample size (40)
Generate a Random start ***R*** by getting a random number between 1 and ***N***

R is the serial number to be selected.

Add the sampling serial number ***K*** to ***R*** to get the second number, then ***K*** to sum and so on to get ***n*** values.

7. Sampling weights

The weights of the sample are in this case equal to the inverse of the product of the two selection probabilities employed (one for each stage of selection).

Therefore, the probability of selecting an EA will be calculated as follows:

$$P^1_{hi} = \frac{a_h M_{hi}}{\sum_i M_{hi}}$$

Where:

P^1_{hi} = the first selection probability of SEAs

a_h = The number of SEAs selected in stratum h

M_{hi} = The measure of size (in terms of the number of households) of the i th SEA in stratum h

$\sum_i M_{hi}$ = The total measure of size of the stratum h

The selection probability of the household will be calculated as follows:

$$P^2_{hi} = \frac{n_{hi}}{N_{hi}}$$

Where:

P^2_{hi} = the second selection probability of selecting households

n_{hi} = the number of households selected from the ith SEA of h stratum

N_{hi} = Total number of households listed in a SEA

Therefore, the SEA specific sample weight will be calculated as follows:

$$W'_{hi} = \frac{1}{P^1_{hi} x P^2_{hi}}$$

All the results in the survey will be weighted. This means that the number of eligible children in each province will be determined within confidence limits.

8. Estimation process of survey parameters

One of the objectives of this survey is to give the estimated number of eligible children in each of the survey strata and at national level. All the estimates in the survey will be weighted.

Therefore, if y_{hij}

is an observations on variable ***Y*** for the ***j***th household (e.g. number of eligible children) in the ***i***th SEA of the ***h***th stratum, then the estimated total for the ***h***th stratum (a particular region) is expressed as follows:

$$Y_{hT} = \sum_{i=1}^{a_h} w_{hi} \sum_{j=1}^{n_h} y_{hij}$$

Where:
Y_{hT} = the estimated total for the hth stratum
i = 1 to a_h: the number of selected clusters in the stratum
j = 1 to n_h: the number of sample households in the stratum

The national estimate is obtained using the following estimator:

Y_T = $\sum Y_{hT}$

Where:

Y_T = the national total estimate

Replacement methodology at each stage

Since the response rate of the households as well as the approximate number of children with eligible children is factored in sample size calculation, the design does not allow for replacements at household level.
